# Information Wanted

**Published:** 1912-09

**Authors:** 


### INFORMATION WANTED

We have often been impressed with the difficulty of obtaining definite information on topics not readily elucidated by reference to dictionaries, cyclopædias, and ordinary text books. Theoretically the published question is the ideal way of getting exact information, of value not only to the inquirer but to many others equally ignorant, who may not even have had their attention called to the particular vacuole. Practically, the Question Box in any kind of a magazine, is liable to degenerate. However, in spite of the warning from the experience of others, we shall, from time to time, publish requests for information, beginning with the following questions, answers to which have not been readily obtained in the regular ways.

1. Temperature. What effect has an air temperature higher than that of the body, upon the mouth temperature? What practical precautions are taken in hot climates to register the correct mouth—or rectal—temperature?

2. Small pox. Is the identity of vaccinia with (modified) variola now generally conceded? What is the modern opinion as to vaccination as a therapeutic means, after variola has begun?

3. Inherited immunity against semelicident infections. These diseases (exanthemata and a few others) very often cause abortion. If not, it is still an open question how far the disease in the mother during pregnancy, protects the offspring. We would be glad of reports of cases showing, on the one hand, resistance to exposure, to vaccination, mitigation of severity of acquired infection; and, on the other hand, cases apparently showing the failure of immunization; also of any observation for or against actual communication of the disease to the foetus.

4. Distribution of infection according to animal species. We have quite a number of notes indicating that certain infections do not occur spontaneously, or even cannot be produced except by exagerated artificial exposure, in certain animals; as well as a miscellaneous series of notes as to the susceptibility of man and other animals to infections. The prevalent notion as to the extreme susceptibility of man, as compared with the lower animals, does not seem to be correct. We would be glad of detailed observations on these points and particularly of general principles as to the range of infectivity according to species, genera, orders and phyla.

5. Chylous accumulations. In preparing as complete a list as possible of chylous cysts of the mesentery, we would be greatly obliged for notes of cases, references to literature, etc.

6. Pioneer Physicians. Published and unpublished bigraphic notes are desired on all American physicians of the seventeenth and eighteenth centuries.

